# Editorial: Advancing mycorrhizal research for sustainable ecosystem and agricultural practices

**DOI:** 10.3389/fmicb.2025.1760087

**Published:** 2026-01-09

**Authors:** Sabine Dagmar Zimmermann, Elisa Taschen, Agnès Robin, Monica Calvo-Polanco

**Affiliations:** 1 IPSiM, Univ Montpellier, CNRS, INRAE, Institut Agro, Montpellier, France; 2 Eco&Sols, Univ Montpellier, CIRAD, INRAE, Institut Agro, IRD, Montpellier, France; 3 CIRAD, UMR Eco&Sols, Montpellier, France; 4 CIALE, University of Salamanca, Salamanca, Spain

**Keywords:** adaption, beneficial interaction, environmental stress, microbiome, mycorrhizal symbiosis, plant nutrition, root, tolerance

Mycorrhizal symbiosis, a mutualistic association between plant roots and soil fungi, plays a crucial role in enhancing plant nutrition, stress tolerance, and overall adaptation to environmental conditions (van der Heijden et al., 2015). Despite the long-standing recognition of these benefits, research has primarily focused on understanding the mechanisms underlying the establishment and functioning of these interactions. Recent studies have highlighted the significant role of fungal hyphal networks in soil carbon storage, further emphasizing the ecological importance of mycorrhizal symbioses. However, there remain substantial gaps in our understanding of how these interactions can be optimized for both natural ecosystems and agricultural applications, especially under the increasing pressure of global climate change.

The present Research Topic (RT) was initiated in the context of the *7*^*th*^ *French Mycorrhizal Days* in Montpellier in May 2024, an international meeting bringing regularly together mycorrhizal research, students and companies from francophone countries (https://jmf7.journees.inrae.fr/). The RT aims to consolidate current research on mycorrhizal symbioses across various levels, from molecular and functional analyses to ecosystem studies and practical applications. The primary objective is to enhance our fundamental knowledge and to facilitate its translation into ecological and sustainable practices for plant growth, soil management, and ecosystem balance. The call for papers attracted several contributions, among which five original papers and one review article were accepted. Key questions regarding mycorrhizal symbioses include the establishment of functional interactions between fungal and plant partners (Klein et al., Ramos-Alvelo et al.) and the impact on plant performance and soil health (Alayafi et al., Battie-Laclau et al.). Potential roles of beneficial microorganisms in mitigating environmental stress linked to climate change are presented (Hassan et al., Lethielleux-Juge).

Mycorrhizal symbiosis is tightly regulated by host plants and fungi and requires major root cell and fungal hyphae reprogramming to form structures enabling nutrient exchange (Choi et al., 2018; Ho-Plágaro and García-Garrido, 2022). Specific molecular players and mechanisms involved in the establishment of arbuscular mycorrhizal (AM) symbioses were investigated in two studies. Strigolactones, phytohormones with dual signalling functions within plant roots and in rhizosphere interactions (Al-Babili and Bouwmeester, 2015), have been demonstrated to impact spore germination and hyphal branching affecting in turn root colonization by AM fungi (Akiyama et al., 2010). Here, Klein et al. analyzed strain-specific impacts of strigolactones using two *Rhizophagus irregularis* strains and their response regarding germination and pre-symbiotic growth. Observed strain-specific differences may suggest a kind of selective management of AM symbiosis dynamics by plant delivered effector molecules. At the plant side, Ramos-Alvelo et al. investigated the tomato α/β-hydrolase SlDLK2, related to the strigolactone receptor D14 and interacting with DELLA (Ho-Plágaro et al., 2021), a negative regulator of arbuscule branching. Transcriptome analysis by RNA-seq of *SlDLK2*-overexpressing tomato roots showed repression of hormone biosynthesis genes, especially carotenoid/apocarotenoid pathways. Overexpression lines displayed strong reductions in jasmonic acid and ABA, and lower auxin and solanacol levels. Comparative transcriptomics confirmed that SlDLK2 downregulates AM-responsive genes, suggesting its role in AM symbiosis inhibition by suppressing hormone pathways that promote it otherwise.

Impact of AM fungi on plant performance was analyzed by Alayafi et al. for two crop plants, sunflower (*Helianthus annuus*) and pumpkin (*Cucurbita pepo*), under field conditions for 2 years. Inoculation with the AM fungus *Funneliformis mosseae* improved plant nutrient uptake, biomass and specifically oil yield and quality, dependent on possible synergistic effects of intercropping (Reddy et al., 2023). Such field studies, going beyond laboratory work under controlled and limited conditions, underline the importance of beneficial interactions for modern agriculture. In turn, Battie-Laclau et al. reported also that the vineyard's “terroir,” understood as a cultivated ecosystem where grapevine interacts with their natural environment, is critical for the composition of the microbiota community. On the other hand, agricultural practices promoting AM fungi in grapevine cultures have been long time neglected as massive fungicide treatments were needed to protect vineyards. Only since recently, with the increasing demand for more natural culture conditions, research is focalizing on promoting AM fungi in vineyards through the use of more organic management practices. Such changes promoting agroecology (Jindo et al., 2022) will induce higher diversity of AM communities and thus determine the future sustainable development of vineyards over next decades. This study showed the dominant effect of geographical effects, AM communities mainly be structured by *terroir* even before practice, underscoring the necessity for future studies on fungal inoculation in vineyards to consider the specific characteristics of each *terroir*.

Another important topic is the impact and use of beneficial interactions for a better adaptation of plants to challenging environmental conditions and climate change. Mycorrhizal symbioses, among other beneficial associations, are playing a decisive role in improving plant tolerance to abiotic and biotic stress (Usman et al., 2021). Regarding other beneficial interactions, Hassan et al. focalized on plant growth-promoting bacteria (PGPB, *Micromonospora* sp) and demonstrated their role in mitigating effects on heat stress in wheat by metabolic adaptations enhancing photosynthetic efficiency and antioxidant defence. The importance of AM symbiosis for the bioavailability of essential micronutrients in wheat has been recently highlighted (Nguyen et al., 2025). In a more general context, Lethielleux-Juge reviewed the impact of mycorrhizal symbioses and associated soil microbiomes in ecological restoration. The use of AM fungi, both natives or exotic, have been widely reported in ecosystem restoration (De Moura et al., 2022). AM fungi grow at diverse soil conditions, colonizing most plant species (from herbs to trees), and can evolve together with plants after revegetation. Moreover, the interaction of AM fungi with different ectomycorrhizal fungi, PGPB, rhizobacteria and mycorrhiza-helper bacteria will allow to improve soil attributes and plant adaptation to stress in a complex network of plant-soil-microbiomes with high relevance in ecosystem dynamics.

In summary, studies included in this RT are dealing with a broad panel of questions regarding our insight in and application of beneficial mycorrhizal interactions. Further questions concern still better understanding of mycorrhizal networks in natural ecosystems, in soil carbon storage, and under diverse challenging abiotic and biotic environmental conditions. For scientifically based agricultural applications, best practices for managing plant-soil biota on the field-scale will have to include whole microbiome interactions.
